# Conventional 22- and 20-gauge needle for second trimester amniocentesis: A comparison of short term outcomes

**DOI:** 10.4274/tjod.74875

**Published:** 2016-03-10

**Authors:** Seyit Ali Köse, Mehmet Özgür Akkurt, And Yavuz, Serenat Eriş, Esra Nur Tola, Mehmet Okan Özkaya, Mekin Sezik

**Affiliations:** 1 Süleyman Demirel University Faculty of Medicine, Department of Obstetrics and Gynecology, Isparta, Turkey

**Keywords:** Amniocentesis, complication, cost-effectiveness, needle size

## Abstract

**Objective:**

To compare the short-term outcomes of two different-sized needles for genetic amniocentesis.

**Materials and Methods:**

A total of 271 amniocentesis were retrospectively evaluated in 2 groups concerning the size of the needles used during the procedure: Conventional 20-gauge (G) (n=164) and 22G (n=107). Periprocedural complications and cost-effectiveness were compared across the groups.

**Results:**

There were no differences between groups concerning complications within 15 days after the procedure (fetal loss, 0.6% versus 0.9%, and amniotic fluid leak 1.2% versus 1.8%, p=0.99 for each). The 22G needle was significantly more cost efficient (p<0.0001).

**Conclusion:**

The 22 G spinal needle is convenient for second trimester amniocentesis with similar complication rate and has a favorable cost profile.

## INTRODUCTION

Amniocentesis is an invasive procedure. Thousands of amniocentesis procedures have been performed for prenatal karyotyping since its first use in 1966^(1)^. Procedure-related complications both during and after amniocentesis have been particularly well-defined, these include amniotic fluid leakage, rupture of chorioamniotic membranes, direct or indirect fetal trauma, infection, and fetal loss, the latter two being the most common complications^(2,3)^.

No single needle type used during amniocentesis has been reported to be associated with lower complication rates^(3)^. Some investigations have compared various needle sizes, periprocedural local anesthetic use, and different techniques of needle use. However, these revealed similar complication rates, especially when different needle sizes were used. Lower procedure-related morbidity seems to be associated with proper selection of pregnancies for the invasive procedure and the experience of the operator performing the amniocentesis^(4)^.

During the present study, we obtained data on a considerable number of amniocentesis procedures concerning different needle sizes in a tertiary perinatology unit. Our aim in this observational study was to compare the complications (including fetal loss, vaginal bleeding, pain, maternal fever, and amniotic fluid leakage), efficacy, and cost effectiveness of two needle sizes used by three professionals for amniocentesis procedures.

## MATERIALS AND METHODS

This observational study was carried out at the Perinatology Unit, Faculty of Medicine, Süleyman Demirel University between January 2012 and December 2014. The study groups included women aged between 24 and 37 years. Two hundred seventy-one pregnant women scheduled for genetic amniocentesis were retrospectively evaluated in the two groups according to the size of the needle used for amniocentesis. Amniocentesis procedures were performed either with a 20-gauge (G) needle specific for amniocentesis and chorion villus sampling (Egemen International Amniocentesis Needle, İzmir, Turkey) (group 1), or with a 22 G spinal needle (Zhejiang Fert Medical Device Co., Ltd. China) (group 2). The participant’s characteristics such as maternal age, parity, and gravidity were recorded. All procedures were conducted in accordance with the Helsinki Declaration. The study protocol was subject to local ethics committee approval. Written informed consent was obtained from all patients who participated in this study.

All women in both arms of the study underwent the standard of care technique under transabdominal sonographic guidance with free hand insertion of the needle into the amniotic cavity^(3,4,5,6,7,8,9,10,11,12,13,14)^. Approximately 1 mL/gestation week of amniotic fluid was aspirated. Data recorded included maternal age, gravidity, parity, history of maternal surgery and the direct cost of the needle used during the procedure. Fetal heart activity was recorded with Doppler ultrasound before and after the procedure. Women were followed up for the development of any complications including vaginal or needle site amniotic fluid leakage, vaginal bleeding, fetal loss, maternal fever, and pain during a 15-day period following the procedure.

Statistical analyses were performed using SPSS version 16.0 software (SPSS Chicago, Il., USA). Student’s t-test, Chi-square contingency table analyses, and Fisher’s exact test were used for comparisons of variables across the groups, with statistical significance set at p<0.05.

## RESULTS

Table 1 lists the demographic characteristics of women participating in the study. A total of 271 women who underwent amniocentesis in our unit over the 3-year period were included.

All women had a singleton pregnancy. Indications for amniocentesis were as follows: increased risk for first and second trimester screening test, advanced maternal age, and family history of genetic disorders. The mean gestational age during the procedure was 17.8±0.9 standard deviation weeks for the whole study group.

There were no significant differences between the groups concerning maternal age, parity, and gestational age at intervention (Table 1). The complication rates of the operators (n=3) were also similar (p>0.05). Abnormal karyotypes were found in 5.8% of the total sample with trisomy 21 being the most commonly detected aberrance. The distribution of abnormal karyotypes did not differ among the two groups (p>0.05).

The number of needle puncture attempts and the volume of collected amniotic fluid did not differ among the two groups. Pain during and/or after the procedure was reported by 10 women (6.1%, group 1) and 9 women (8.4%, group 2), respectively (p=0.627). Amniotic fluid leakage was observed in 4 women, 2 in each group. There was no significant difference between the groups according to maternal fever and vaginal bleeding. A total of 2 fetal losses occurred within 2 weeks of amniocenteses (1 in each group). Therefore, complication rates were similar (Table 2). However, cost was significantly lower with the 22 G spinal needle (14 USD for 20G needle versus 0.53 USD for 22 G needle, p<0.0001).

## DISCUSSION

Patient comfort and safety as well as cost effectiveness are important issues for amniocentesis. Cost-effectiveness might be a concern because the procedure is performed quite frequently and laboratory cost for karyotyping is relatively high. Therefore, needle size, length, and ultrasonographic visibility would be expected to have an effect on quality, comfort, and cost-effectiveness of this invasive procedure. Although use of needles with lower sizes were considered to be associated with less trauma and complications, previous studies did not support such theoretical statements; no extra advantage was provided with different-size needles^(2,7)^.

Previous investigations have included comparisons of various needle sizes, needles with improved ultrasound visibility characteristics, and simple large-bore spinal needles^(2,5)^. However, cost effectiveness for different needle sizes has not been investigated. In the current study, we expanded previous data by including cost effectiveness in our design.

Previous publications compared various parameters including post-procedure complications among needles of different sizes used during amniocentesis. These previous data revealed that different needles were generally comparable with no significant differences concerning morbidity^(2,6,7)^. Small-sized needles have been associated with certain difficulties during the procedure. These include longer amniotic fluid aspiration times, primarily due to clogging and requirement for a second administration^(8)^. Our clinical experience with the 22 G spinal needle was somewhat similar, indicating longer aspiration time, clogging due to particulation, and requirement for further punctures; however, the retrospective design of our study did not allow for measurements of the exact aspiration time. Devlieger et al.^(9)^ reported similar mean duration of sampling among all operators. However, when experience was considered, expert operators showed shorter sampling times with a 22 G versus 23 G needle.

One of the most devastating complications of amniocentesis is fetal loss. Recent data have indicated experience as the most important factor in effecting fetal loss^(10,11)^. Large-bore needles might be associated with relatively easier puncture and fluid aspiration as well as shorter interval and can facilitate procedures that require transplacental passage^(12)^. One would expect large-bore needles to lead to increased intrauterine bleeding following transplacental passage^(11,13)^. However, this is not supported by at least one study that reported more intrauterine (intra-amniotic) bleeding with needles of smaller size^(2)^. Moreover, investigations that included very small-gauged needles such as 29 G reported difficulties during myometrial passage and membrane puncture as the primary drawback^(8,14)^. Although there are some data on the cost effectiveness of karyotype analysis, needle costs have not been included in previous studies^(12)^. Proper indication, informed consent, and experienced operator decrease fetal loss rates of amniocentesis procedures^(10)^. Single amniotic entry and decreased procedure time also decrease maternal anxiety.

## CONCLUSION

The exception of cost, our data from a single center revealed similar outcomes with needles of two different sizes used during amniocentesis procedures by theree different operators. Therefore, 22 G needles, which are commonly used during spinal anesthetic administration, may also be suitable for performing amniocenteses.

## Figures and Tables

**Table 1:**
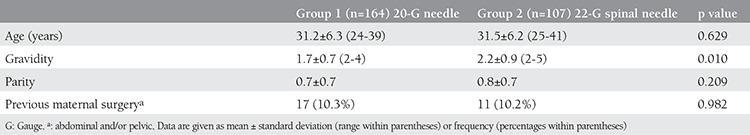
The demographic data of the pregnant women in study group

**Table 2:**
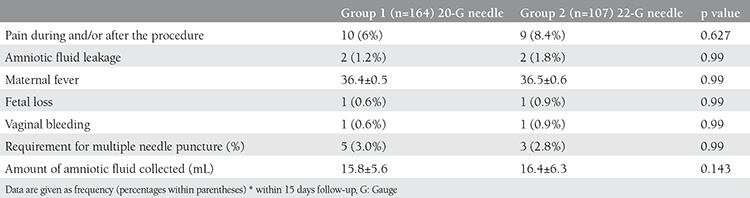
Complications during and after the procedures*
